# Use of Saliva for Diagnosis and Monitoring the SARS-CoV-2: A General Perspective

**DOI:** 10.3390/jcm9051491

**Published:** 2020-05-15

**Authors:** Jose J. Ceron, Elsa Lamy, Silvia Martinez-Subiela, Pia Lopez-Jornet, Fernando Capela-Silva, Peter David Eckersall, Asta Tvarijonaviciute

**Affiliations:** 1 Interdisciplinary Laboratory of Clinical Analysis, Interlab-UMU, Regional Campus of International Excellence ‘Campus Mare Nostrum’, University of Murcia, Espinardo, 30100 Murcia, Spain; silviams@um.es (S.M.-S.); asta@um.es (A.T.); 2 Mediterranean Institute for Agriculture, Environment and Development (MED), Advanced Research and Training Institute (IIFA), University of Évora, 7006-554 Évora, Portugal; ecsl@uevora.pt; 3 Department Stomatology School of Medicine, Biomedical Research Institute (IMIB-Arrixaca), Faculty of Medicine and Odontology, University of Murcia, 30008 Adv Marques de los Velez s/n, Spain; majornet@um.es; 4 Department of Biology, School of Sciences and Technology. Mediterranean Institute for Agriculture, Environment and Development (MED). University of Évora, 7006-554 Évora, Portugal; fcs@uevora.pt; 5 Institute of Biodiversity, Animal Health and Comparative Medicine, University of Glasgow, Bearsden Rd, Glasgow G61 1QH, UK; david.eckersall@glasgow.ac.uk

**Keywords:** saliva, coronavirus, SARS, Covid-19

## Abstract

In this report, updated information and future perspectives about the use of saliva as a sample for laboratory analysis of the Covid-19 are highlighted. Saliva can be used for the direct detection of the SARS-CoV-2 virus, the quantification of the specific immunoglobulins produced against it, and for the evaluation of the non-specific, innate immune response of the patient. Moreover, a deeper knowledge of potential changes in the saliva proteome in this disease may allow the identification of new diagnostic and prognostic biomarkers, or even help our understanding of the mechanisms associated with the disease. With the development of appropriate sample collection and processing methods and the use of adequate assays, saliva can provide useful clinical information about the disease and could be potentially included in guidelines for sample collection for the diagnosis, disease management, and control of Covid-19.

## 1. Introduction

A coronavirus, designated by the World Health Organization as 2019 novel Coronavirus (2019-nCoV) and by the Coronavirus Study Group of the International Committee on Taxonomy of Viruses as severe acute respiratory syndrome coronavirus 2 (SARS-CoV-2), has been spreading worldwide, producing the coronavirus disease 2019 (Covid-19). Although its mechanism of infection is still not known, it has affinity for and replicates in cells located in the lower airways. The most characteristic clinical sign is the production of a respiratory distress syndrome that can range from mild malaise to death, having worse prognosis in elderly patients with comorbidities [1]. In addition, one of the symptoms, referred by many patients, is the loss of sensory acuity, including smell and taste [2]. On the 18th of April, the number of cases worldwide was 2,261,034 with 154,726 deaths.

Saliva has been increasingly used over the last few decades for evaluating human health. It is an integrated mixture of secretions of the different salivary glands, desquamated oral epithelial cells, gingival crevicular fluid, and different microorganisms [3]. It also contains a large number of proteins such as immunoglobulins, mucins, and enzymes, as well as metabolites, hormones, and electrolytes. This composition allows the detection of pathogens in saliva and also the quantification of biomarkers that can provide information about the immunological, inflammatory, endocrine, and metabolic status of the individual. In some cases, its value to detect physiological changes is similar or superior to serum, such as for the detection of acute stress by alpha-amylase or cortisol [4]. Overall, saliva appears to be a fluid of enormous potential in health assessment, especially due to the clinical information that it can provide and the non-invasive nature of its collection, which can be performed by individuals without particular training and with no major requirements in terms of equipment or facilities.

Currently, naso and oropharyngeal swabs are the two main recommended upper respiratory tract specimen types for Covid-19 diagnostic testing, although the use of saliva for the diagnosis of the disease has been recently suggested [5,6]. Furthermore, in recent days, a test for assessing the RNA in saliva samples was approved by the US Food and Drug Administration [7].

The objective of this report is to provide updated information about the use of saliva as a sample for laboratory analysis in Covid-19 investigations. For this purpose, the advantages of the use of this biofluid are described and the scientific evidence is presented, which supports the concept that the analysis of saliva in patients with Covid-19 could allow for the detection of the virus and antibodies produced against it, as well as the assessment of the non-specific, innate immune response. Finally, the possibility of the use of saliva for finding new biomarkers in this disease and some general recommendations that could contribute to a more appropriate use of this sample will be highlighted.

## 2. Advantages of the Use of Saliva

The use of naso and oropharyngeal swabs have several limitations, such as the discomfort for the patient and the need for the intervention of a healthcare worker in a disease with a high risk of nosocomial transmission [8]. These collection systems can also induce coughing and sneezing, generating aerosol, which can transmit the virus. In addition, in cases of thrombocytopenia or any other coagulation disorder, this procedure can cause bleeding. These drawbacks can limit the use of swabs, especially in serial monitoring or mass test programs. Sputum has been also proposed as a non-invasive lower respiratory tract specimen, but 72% of Covid-19 patients were not able to produce it for collection [9]. The difficulty of obtaining sputum also has been described in SARS-CoV, a virus with many similarities with the Covid-2019, especially at early stages of infection, when no cough or only dry cough is present [10]. The use of saliva could improve these drawbacks as it has the following advantages:

- It can be collected by the patient, even at home, minimizing the exposure of health care workers to nosocomial infections. This also reduces the need for health care personnel and waiting times for sample collection, resulting in easier crowd control regulations in clinical settings and thus avoiding further virus transmission.
- It is easily accepted by the patients since it is non-painful and non-stressful. Therefore, it can be used for serial samplings and in large scale or epidemiological studies, being especially advantageous in certain populations, such as children [11].
- It is easy, fast, and cheap to collect, allowing widespread testing.

**Figure 1:**
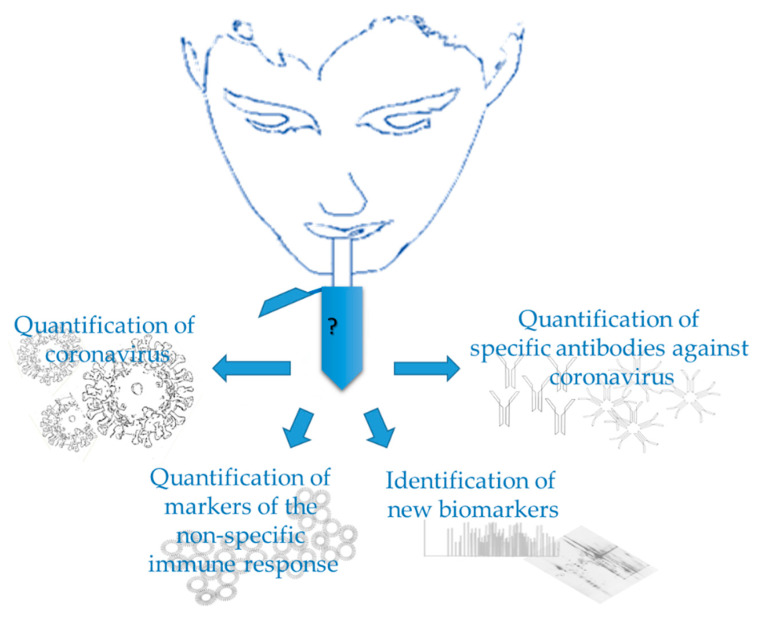
Use of saliva in Covid-19.

In the particular case of SARS-CoV-2, saliva could be used for evaluating different aspects of the disease, as shown in Figure 1, the evidence for which will be described in the following sections.

## 3. Quantification of Coronavirus in Saliva

### 3.1. Evidence for SARS-CoV-2 in Saliva

Currently there are publications from three different research groups that have used saliva for SARS-CoV-2 detection:

- The group of Dr. To published two reports using saliva collected by asking the patient to cough out saliva from their throat into a sterile container and adding a viral transport medium to the sample [12,13]. These samples, in addition to salivary gland secretions, contained material from the posterior oropharynx, that could have come from respiratory secretions swept up from the tracheal–bronchial tree, and also secretions coming down from the nasopharynx.
  In these reports, the RNA of the virus was identified in the saliva of 20 out of 23 patients who had previously been confirmed as infected by the detection of SARS-CoV-2 RNA in their nasopharyngeal or sputum specimens, giving an overall diagnostic sensitivity of 87%. In addition, saliva tested negative in 33 patients from whom nasopharyngeal specimens were negative for SARS-Cov-2. The detection of the virus was by a real-time reverse transcription-quantitative polymerase chain reaction (rRT-PCR) and the range of values was from 9.9 × 10^2^ copies/mL to 1.2 × 10^8^ copies/mL.
- Azzi et al. [14] collected saliva through passive drool. In cases of patients who were undergoing endotracheal intubation and mechanical ventilation, the collection was performed intraorally by a physician with the use of a pipette. These specimens possibly also contained respiratory secretions. In these conditions, SARS-CoV-2 was detected in all saliva samples collected from a group of 25 patients with severe to very severe disease, who were diagnosed by detection of the virus in pharyngeal or bronchoalveolar swabs. An rRT-PCR that detected a trend in viral load without quantification of the viral copies per millimeter, was used for virus detection.
  In this study, two patients who were monitored showed positive salivary results on the same days that their pharyngeal or bronchoalveolar swabs were negative. This raises the possibility that individuals can be contagious through their saliva even when pharyngeal swabs are negative. This could be a point in favor of the use of saliva for the virus detection and would be in line with the description of salivary glands as potential reservoirs for Covid-19 in asymptomatic but infected people [15].
- Han et al. [16] collected saliva from a 27-day-old neonate diagnosed with Covid-19 and reported values in the range of 10^5^ copies/mL that were similar to the values obtained with pharyngeal swabs but lower than those from bronchoalveolar swabs.

### 3.2. Evidence from the Previous SARS-CoV Epidemic

SARS-CoV was detected in the saliva of 14 SARS patients at high amounts, ranging from 7.08 × 10^3^ copies/mL to 6.38 × 10^8^ copies/mL [10]. In the same study, throat wash by gargling 10 mL normal saline was collected in an airborne isolation room from all these patients and the supernatant was analyzed after centrifugation. The amount of SARS-CoV RNA in the saliva was greater than in the throat wash, with values in the throat wash ranging from 9.58 × 10^2^ copies/mL to 5.93 × 10^6^ copies/mL [10]. This high detection rate appeared at a median of four days after disease onset and before the appearance of lung lesions. In another study, lower values (5.2–5.5 × 10^2^ copies/mL) were obtained in the saliva of three patients with SARS-CoV [17]. These divergent results could be influenced by the differences in the methods used and samples collected. 

In an in vivo experiment in rhesus macaques, after intranasal inoculations with a pathogenic SARS-CoV, the angiotensin-converting enzyme-2 receptor positive epithelial cells lining the salivary gland ducts were target cells, being infected at an early stage [18]. SARS-CoV was readily detected in oral swabs in four infected macaques, but in only two of them was the virus detected in lungs and it was not detected in blood in any animal. This experiment suggests that the virus appears in saliva at an earlier stage of infection than in lungs and could reach saliva due to direct salivary gland infection.

### 3.3. Evidence from Other Coronavirus and Virus in General

The group of Dr. To, using saliva samples obtained by the expectoration of saliva into a sterile container, has reported a high concordance (higher than 90%) between saliva and nasopharyngeal specimens in the detection of coronavirus and other respiratory viruses, and in some patients, coronavirus was detected only in saliva but not in nasopharyngeal aspirate. In addition, they indicated that saliva can be used for the detection of patients who were subclinically infected with respiratory viruses. The authors indicated that these collected saliva could be mixed with sputum because it was obtained by expectoration [19]. In animals, the RNA of the coronavirus, called Porcine epidemic diarrhea virus (PEDV), can be detected in the saliva of infected pigs by real-time PCR at the same time but at higher concentrations than in serum [20,21]. In other species of virus such as the flavivirus Zika, the virus can be detected in the saliva of infected human patients with higher sensitivity than in serum [22]. In addition, saliva is widely used in the diagnosis of cytomegalovirus infection [23].

## 4. Quantification of Specific Antibodies against Virus in Saliva

Another field where saliva may have a major potential for Covid-19 is in screening for immunity. There is scientific evidence that specific antibodies against infectious diseases can be detected in saliva [24]. Salivary IgG and IgM concentrations are much lower than in serum, although it has been suggested that they are in a similar proportion in relation to total protein in saliva as in serum [25]. In the particular case of IgG, plasma and saliva IgG profiles are highly similar for a large number of antigens [26]. It has been hypothesized that both salivary IgG and IgM are derived from blood, whereas IgA is mainly produced by the salivary glands [24].

The presence of IgM and IgG in serum against components of the virus, such as 2019-nCovid nucleoprotein (NP) and spike protein receptor binding domain (RBD), has been described in Covid-19 patients 10 days or later after symptom onset [12]. Although, to the author’s knowledge, there are presently no studies regarding antibodies in saliva in Covid-19. In animal species, there is evidence that saliva can be used for the assessment of antibody responses to coronavirus infections. For example, saliva IgG and IgA can be used to track levels of immunity against PEDV disease over time [20]. In addition, saliva has been used in several viral diseases of humans for the detection of specific immunoglobulins to pathogens such as hepatitis A, B, and C, human immunodeficiency virus, and rubella virus [24,27,28].

The detection of antibodies in saliva could be potentially used for the control of Covid-19 as occurred with other infectious diseases. For example, for infection with rubella, oral fluid testing has been offered by the Public Health Laboratory Service in the UK for cases notified to the Office for National Statistics (ONS). In this program, saliva samples are collected between two and six weeks after the onset of symptoms and tested for virus specific IgM. The assays used have been shown to be greater than 90% sensitive and specific [25]. It could be expected that saliva will have similar value in Covid-19 diagnosis and monitoring. This is particularly interesting for two main reasons: (1) as long as there is no effective treatment or vaccine and person-to-person transmission has to be minimized, the possibility of identifying immune individuals will be invaluable in defining who and how particular individuals can be relieved from confinement and limited social contact; (2) there is a lot to establish and understand about immunity dynamics during the disease, and how long it remains after the individual has recovered [29].

## 5. Quantification of Markers of the Non-Specific Immune Response in Saliva

In patients with Covid-19, serum concentrations of acute phase proteins (APPs), such as C-reactive protein (CRP) and ferritin, are increased in the cases that develop more severe disease. Their concentrations have been shown to correlate with the severity of the process [30,31]. In inflammatory conditions, APPs can increase before the appearance of clinical signs, being very early biomarkers [32]. In addition, increases in serum of several interleukins (IL), such as IL-6 and IL-10, have been described in Covid-19 patients [33], and these cytokines are known to be mediators of the APPs response.

In humans and in animal models, a high correlation in CRP between serum and saliva has been demonstrated [11,34]. Additionally, other APPs such as ferritin, haptoglobin, serum amyloid A, different interleukins, and other analytes related to the immune response, such as adenosine deaminase (ADA), can be measured in saliva [11,35,36].

These analytes can potentially be used as salivary biomarkers to assess the severity of the process and also to predict the development of more severe cases in Covid-19.

## 6. Saliva as a Source for New Disease Biomarkers and/or Understanding of Pathways Involved in Disease

The use of proteomic techniques allows the identification of multiple proteins in saliva [37]. By comparing these proteins between healthy individuals and those with disease, it is possible to assess the differences, which can result from changes in the circulating levels of proteins and/or from changes in the salivary gland secretion, associated with a disease. These differences are valuable to understand the pathophysiological processes associated with the disease, and identify biomarkers that may allow an early and easy diagnosis, and have been studied in other viral diseases [38].

In Covid-19, proteomic studies could allow for the discovery of new biomarkers for the disease and help to elucidate the loss of smell and taste by some patients [39]. It is known that the saliva proteome is related to taste sensitivity [40]. In this context, potential changes in the saliva proteome in this disease may be associated with the reduced taste perception.

## 7. Recommendations and Points to Improve for an Optimal Use of Saliva as Sample

Although saliva is a sample easy to collect, general recommendations should be followed for its use, some of which are particularly focused in the application to this disease, as shown in Table 1.

1. The use of a standardized method of collection.

Different methods can be used for saliva collection, such as passive drool, different absorbent materials, or stimulation with citrate. These methods may have, in some cases, interference with selected analytes. In the particular case of the Covid-19, two aspects should be addressed regarding the method of collection:

-The possible differences between saliva obtained by passive drool or absorbent materials and by clearing the throat should be evaluated. In addition, some patients might not be able to clear the throat effectively to cough out saliva from deep in the throat and, therefore, this could decrease test sensitivity with this collection method, which should be explored. Although there are no studies comparing the use of saliva with throat wash in Covid-19, in the previous SARS-CoV epidemic similar results were obtained when throat wash with 10 mL of saline and saliva were compared [10]. 

-Instead of direct spitting, the use of a straw or any device that avoids the creation and expansion of drops should be encouraged. The saliva of infected patients can contain viruses that may allow airborne transmission and also by oral droplets and, therefore, should be handled with care [14]. In SARS-CoV, it has been hypothesized that very small particles of the virus can be spread in the air [10]. Therefore, special care should be taken during all the processes of sample collection, management, and analysis to eliminate this potential risk of transmission via contact with saliva droplets or aerosol.

Taking these facts into consideration, preliminary tests should ideally be made to establish the optimal method of collection, which may be different depending on the analyte to be measured. For example, in Covid-19, it would be of interest to evaluate if the saliva obtained by clearing the throat could be more convenient for the detection of the virus, whereas the whole saliva obtained directly by drool could be more suitable for the quantification of antibodies or acute phase proteins.

In addition, ideally, the salivary flow should be controlled by establishing a fixed amount of time during which saliva is collected (e.g., one minute). It is worthwhile to indicate that the report of results should be standardized, since in some cases, such as for selected interleukins or alpha-amylase, the way of expressing the values can influence their interpretation [4]. Furthermore, the need for saliva sample centrifugation and the effect of this centrifugation on the analyte of interest should be assessed. Avoiding centrifugation can speed the sample processing, allowing the use of rapid point of care tests; however, centrifugation helps to eliminate components that can interfere with subsequent assays. 

2. Use of appropriate conditions for sample preservation. 

It is known that the degradation of saliva components can occur under certain storage conditions [41]. As such, it is important to know the best storage conditions for samples that allow the maximum preservation of the analytes to be analyzed. Nevertheless, while such data are lacking, it is recommended to keep samples refrigerated until arrival at the lab to be analyzed and use −80 °C temperatures if the samples are stored.

3. Use of appropriate assays. 

Ideally, the assays used should be sensitive enough for detecting low amounts of the analyte that can appear in saliva. In this line, highly sensitive immunoassays, such as those based on time-resolved fluorescence or similar technologies, would be recommended for immunoglobulins and acute phase proteins quantification [25]. In the case of qRT-PCR, it would be of importance to select an appropriate housekeeping gene and explore if the virus detected in saliva is cell-free or cell-associated. Overall, assays should be fully validated in order to avoid analytical errors due to assay technical limitations.

4. Increase the knowledge base on clinical applications. 

Further studies should be made to evaluate and refine the interpretation and clinical application in Covid-19 of the different analytes that can be measured in saliva. These studies should be focused on their use in different clinical aspects such as in diagnosis, evaluation of disease severity, and in monitoring the response to treatment and predict relapses. 

As an example, studies on the kinetics of antibodies in saliva compared to serum could be undertaken. In some diseases, a delay in the appearance of antibodies in saliva compared to serum exists and should be taken in consideration for an appropriate interpretation of the test [42].

## 8. Conclusions

Saliva can have potential applications in the context of Covid-19 by direct detection of the virus, quantification of the specific immunoglobulins produced against it, and for the evaluation of the non-specific, innate immune response of the patient. Moreover, a deeper knowledge about potential changes in the saliva proteome may allow for the identification of new diagnostic biomarkers or help to understand the mechanisms associated with the disease. With the development of appropriate sample collection and processing methods and the use of adequate assays, saliva can provide useful clinical information about the disease and could be potentially included in guidelines for sample collection for the diagnosis, disease management, and control of Covid-19. 

## Figures and Tables

**Table 1:** Recommendations for the use of saliva as sample in Covid-19.

Use a standardized method for saliva collection, which minimizes the potential risk of transmission via contact by saliva droplets or aerosol. Use appropriate conditions for sample preservation. Use appropriate assays, validated and with sufficient sensitivity for application in saliva. Increase the knowledge base on clinical applications, allowing for a more accurate interpretation of the results.
